# Perceived facilitators and predictors of positive change and posttraumatic growth following a first episode of psychosis: a mixed methods study using a convergent design

**DOI:** 10.1186/s12888-020-02693-y

**Published:** 2020-06-08

**Authors:** Gerald Jordan, Ashok Malla, Srividya N. Iyer

**Affiliations:** 1 ACCESS Open Minds/Esprits ouverts, Verdun, Quebec Canada; 2 Department of Psychiatry, McGill University, 6875 Boulevard LaSalle, Verdun, Montreal, Quebec H4H 1R3 Canada; 3 Yale Program for Recovery and Community Health, Yale University, New Haven, CT USA; 4 Prevention and Early Intervention Program for Psychoses, Verdun, Quebec Canada

**Keywords:** Posttraumatic growth, Positive change, Recovery, First episode psychosis, Schizophrenia spectrum disorders

## Abstract

**Background:**

This study aimed to identify predictors and perceived facilitators of positive change and posttraumatic growth in persons with a first episode of psychosis using a mixed methods convergent design.

**Methods:**

In the quantitative component, 94 participants completed measures of posttraumatic growth and predictors of posttraumatic growth. The qualitative component involved in-depth interviews with 12 participants.

**Results:**

Quantitative results revealed that being hospitalized for psychosis, spiritual coping, positive reframing and subjective recovery were significant predictors of posttraumatic growth. Qualitative findings revealed that positive change was perceived to be facilitated by the psychosis itself; receiving mental health services; drawing on personal and social resources and strategies; healing and recovering; a meaning-making and knowledge gaining process; and normative developmental processes.

**Conclusions:**

Posttraumatic growth following a first episode of psychosis may therefore be facilitated by complex person-environment interactions.

## Background

A first episode of psychosis is the time when symptoms associated with psychotic illnesses first emerge, and include both positive symptoms (e.g., hallucinations, delusions, etc.) and negative symptoms (e.g., affective flattening, loss of motivation, etc.). First-episode psychosis (FEP) is often a traumatic experience [1] that typically disrupts important life trajectories of young adults. The negative sequelae of psychosis are well documented, and include disruptions to relationships, feeling hopeless, and losing one’s sense of self [2]. However, just as positive changes can occur following traumatic “physical” illnesses (e.g., HIV) [3], positive changes can also follow a first episode of psychosis [4]. The most well-established model of positive change is that of posttraumatic growth (PTG), defined as positive changes that follow the struggle with adversity. Domains of PTG include a stronger sense of self, improved relationships with others, and a greater appreciation of life following adversity [5]. Quantitative results from a recent mixed methods study showed that following FEP, participants were likely to report moderate amounts of PTG. Qualitative findings from this study showed that in addition to suffering, participants experienced positive change at the individual-level (e.g., a strengthening of the self and personality and improved health), spiritual-level (e.g., a stronger connection to God), interpersonal-level (e.g., improved relationships with others), and lifestyle-level (e.g., better lifestyles, goals, and expectations) [6].

PTG following negative experiences are facilitated by a range of factors and processes. For instance, negative experiences have a curvilinear relationship with PTG: experiences that have moderate impact are more likely to lead to PTG, while adversities that have a minimal or highly severe impact are less likely to lead to PTG [7]. Engaging in positive reframing (i.e., seeing good in the bad), spiritual coping and receiving social support are robust predictors of PTG [8]. Trait resilience (i.e., one’s general ability to bounce back from stress) has also been linked to PTG [9]. Other scholars have theorized how resilience comprises resources that persons can draw on to negotiate adversity, and their capacity to obtain those resources [10], and may thus facilitate PTG. Finally, experiencing personal recovery from a FEP may also be linked to PTG [4].

Studying positive change following a first episode of psychosis may be meaningful given the lack of hope young people often feel in its aftermath. Knowing more about how young people can change for the better, and the processes that enable such change, can help mental health services better deliver strengths-based, recovery-oriented care. However, only one quantitative study has investigated *predictors* of PTG following FEP to date [11]. This study reported that recovery and self-disclosure predicted PTG; however, it relied on a small sample (*n* = 34), thus limiting the generalizability of the findings. In addition, only one qualitative study has described processes leading to PTG following FEP [12], and showed that PTG emerged through determining “how psychosis fits into my story”, “breaking free from psychosis” and “fighting my way through psychosis”. Hence, there is a need for additional research on facilitators of PTG following psychosis. Mixed methods approaches may be particularly valuable in this regard given their potential for quantitively and qualitatively examining the role of specific facilitators of PTG (e.g., coping styles) and unpacking their subjective meaning.

To address this knowledge gap, our study posed two research questions:

- Qualitative Research Questions: 1) What are the aspects of PTG service users experience following a FEP 2) What do service users perceive as facilitating aspects of PTG following FEP?
- Quantitative Research Questions: 1) What aspects of PTG are most frequently endorsed by service users following FEP? 2) Which factors predict PTG following a FEP?

## Methods

### Study setting

Participants were recruited from the Prevention and Early Intervention Program for Psychoses. This is the only early intervention service for FEP in Southwest Montreal, Canada, and serves approximately 400,000 people. Services are delivered by a multidisciplinary team consisting of case managers hailing from different professions (e.g., social work, occupational therapy, etc), psychiatrists and psychologists. Eligibility criteria for entry into the program include being between the ages of 14 and 35; having an IQ of at least 70; experiencing a non-affective or affective psychosis unrelated to an organic brain disorder or exclusively explained by substance use; and not having taken antipsychotic medication for more than 30 days [13].

### Study eligibility criteria

To participate in the quantitative and qualitative component, potential participants had to have been in treatment for at least 6 months; be fluent in either English or French; and be between the ages of 18 and 40. In addition, they had to be identified as clinically stable by their treatment team (i.e., not currently experiencing a relapse). We defined relapse as an exacerbation of positive symptoms that necessitated a change in treatment and/or hospitalization. Positive symptoms were assessed by the treating team during regular follow-up meetings and often supplemented by the administration of structured psychiatric rating scales such as the Scale for the Assessment of Positive Symptoms, which is part of the protocol at this early intervention service for psychosis (Iyer et al., 2015).

Further, potential participants were approached for the qualitative component once they were identified by their treatment team as having experienced positive change following FEP. For instance, service users were referred to the qualitative component if they had mentioned that they had experienced positive change following the psychosis during discussions with their treatment team.

### Overall methods and data collection

As per our protocol, [14], this study employed a mixed methods convergent design, whereby both qualitative and quantitative components were conducted concurrently and mixed at the level of research questions, data collection, and interpretation. Using a convergent design allowed us to capitalize on the strengths of both methods [15] and address pertinent knowledge gaps in the qualitative and quantitative research.

### Pragmatic stance

Dialectical pluralism, a metaparadigm that helps researchers thrive off tensions inherent in using multiple methods and paradigms [16], was the overarching paradigm guiding this study. Specifically, the quantitative component was guided by a post-positivist paradigm (which acknowledges the existence of a measurable objective reality that is nonetheless influenced by subjective perceptions) while the qualitative component was guided by a constructivist paradigm (which acknowledges the existence of multiple truths and interpretations of reality) [17].

### Quantitative methods

The quantitative component employed a cross-sectional survey design, whereby a sample of participants completed questionnaires assessing PTG and predictors of PTG at one time-point. A power analysis determined the sample size needed to achieve a moderate effect size with 80% power, and to allow the regression of five independent variables on one dependent variable [14]. Questionnaires were completed in English (*n* = 62) or French (*n* = 32).

### Qualitative methods

The qualitative component was guided by a qualitative descriptive methodology [18, 19]. Data were collected using semi-structured interviews held in English and lasting approximately 1 hr (*M* = 46 min, *Range* = 26–65 min). Participants were purposefully sampled until no new information was obtained. An interview guide developed by the authors in consultation with multiple stakeholders (i.e., clinicians, researchers, service users, families) was used to probe for the reasons why participants felt they received treatment at the early intervention service; how participants changed as a result of these reasons; and participants’ perceptions of what facilitated such changes. The guide also contained probes to elicit perspectives on how participants coped with FEP; the social support they received; the meaning of recovery and where participants felt they were in the recovery process; as well as participants’ connection to culture and community. The interviewer remained open to discussing additional areas that participants felt were important to the topic under investigation.

The first author wrote reflections after each interview. He also engaged in reflexive practice to examine how his multiple brought selves may have shaped the study findings [20]. The specific “selves” that were reflected upon included the first authors’ *brought self*, *situationally created self, and research-based self*. For instance, by reflecting upon his *brought self,* he reflected on his views of spirituality and religion as an atheist to ensure he would not devalue participants’ spiritual/religious experiences described during interviews. In reflecting on his situationally created self, he sought to ensure that information about participants presented during clinical rounds were “blocked off” in order to fully honour participants’ subjective experiences. In reflecting upon his *research-based self*, he reflected on the challenges encountered in using multiple methods and paradigms. He did this by fully engaging with either quantitative or qualitative material at different time points, rather than partially engage with both types of methods simultaneously, in order to maintain consistency of thought.

### Ethics

The study was approved by the McGill University Ethics Board. Written informed consent was obtained from all participants.

### Measures

#### Main predictors

Well-validated, widely used measures were chosen for this study. Positive change was measured using the PTG Inventory [21], which includes 21 items rated on a 6-point Likert-type scale across five domains, namely, appreciation for life (e.g., I have a greater appreciation for the value of my own life); relating to others (e.g., I can more clearly see that I can count on people in times of trouble); spiritual change (e.g., I have a better understanding of spiritual matters); new possibilities (e.g., new opportunities are available which wouldn’t have been otherwise); and personal strength (e.g., I know better that I can handle difficulties). When completing the scale, participants were asked to rate how they changed as a result of their “mental health problem”.

The impact of psychosis was measured using the Impact of Experiences subscale of the Subjective Experiences of Psychosis Scale [22], which consists of 29 items rated on two dimensions: negative impact and positive impact. Participants were asked to describe the mental health problem for which they were being treated at the early intervention service. They then indicated both the negative and positive impact of their mental health problem on various aspects of their lives (e.g., energy, etc.), resulting in a total negative impact score and a total positive impact score. Each item was rated on a 5-point Likert-type scale (not at all to very much). Given that the literature has identified how the negative impact of experiences predict PTG, only the negative impact score derived from the Impact of Experiences subscale was used.

The “situational” version of the Brief COPE scale was used to measure the ways that participants coped with FEP [23]. Participants rated how they coped with their “mental health problem” on a 28-item, 4-point Likert-type scale (“I haven’t been doing this at all” to “I’ve been doing this a lot”). Only the positive reframing (e.g., I’ve been looking for something good in is happening) and spiritual coping (e.g., I’ve been praying or meditating) subscales were analyzed.

Perceived social support was measured using the Multidimensional Scale of Perceived Social Support [24]. The scale comprises 12 items rated on a 7-point Likert-type scale and assesses support from family (e.g., my family really tries to help me), friends (e.g., I can count on my friends when things go wrong) and a special person (e.g., there is a special person in my life who cares about my feelings).

Given the developmental specificity of measures of resilience, we used the youth and adult versions of one resilience measure developed by the same team using a similar conceptual framework. We chose this measure because of its well-established psychometric properties [25] and because it measures resilience as personally and ecologically based, which we theorized could contribute to PTG following psychosis. Participants aged 18 to 23 completed the Brief Child and Youth Resilience Measure, while participants who were 24 years of age or older completed the Brief Adult Resilience Measure [25]. Both measures assess the presence of different aspects of resilience on a 12-item, 3-point scale (no, sometimes and yes). Areas measured are education (e.g., I feel I belong at school); personal skills (e.g., I try to finish what I start); peer support (e.g., I think my friends care about me when times are hard); social skills (e.g., I know where to go in my community to get help); caregiver support (e.g., I feel my parents/caregivers know a lot about me); and connection with culture (e.g., I like the way my community celebrates things). Both versions measure the same domains, with slight variations in wording (e.g., I have people I want to be like vs. I have people in my life who I can respect), and in what is included in each domain (e.g., getting an education is important to me vs. getting and improving qualifications and skills is important to me).

Recovery was measured using the Recovery Assessment Scale [26], which contains 41 items rated on a five-point Likert-type scale (strongly disagree to strongly agree) across five domains, including personal confidence and hope (e.g., fear doesn’t stop me from living the way I want to); willingness to ask for help (e.g., I know when to ask for help); goals and success orientation (e.g., I have my own plan for how to stay or become well); reliance on others (e.g., even when I don’t believe in myself, other people do); and a lack of domination by symptoms (e.g., symptoms interfere less and less with my life).

Validated versions of French questionnaires were used when available. When French versions of questionnaires were unavailable, English questionnaires were translated into French using a well-established method [27]. Specifically, we translated the PTG Inventory [21], the Subjective Experiences of Psychosis Scale [22] and both resilience measures [25]. All questionnaires were pilot tested to ensure their readability.

#### Covariates

We chose to assess certain covariates informed by a review of the literature on predictors of PTG and on key aspects in FEP. We assessed and included age because a younger age has been associated with PTG [28]. We included sex because females may be more likely than males to experience PTG [29]. We included the time elapsed between when participants’ were diagnosed and when they completed the study questionnaires, since some studies have shown that the time elapsed since an adversity is related to PTG [30]. We included positive and negative symptoms of psychosis because a previous study found that these symptoms were related to PTG in a multiple-episode psychosis sample [31]. Finally, we included being hospitalized for FEP since hospitalization may be perceived as a significant and traumatic life event that may nonetheless foster PTG [32]. Age and gender were recorded using a demographic questionnaire completed by participants themselves. Data on other covariates were collected by trained research staff with high inter-rater reliability [13].

Symptoms were assessed at multiple time points during all participants’ follow-up (baseline, months 1, 2, 3, 6, 9, 12, 18, and 24, and every 3 months after month 24 for participants who received more than 2 years of follow-up). The symptom assessment closest in time to when study questionnaires were administered was used.

Positive and negative symptoms were measured using the Scale for the Assessment of Positive Symptoms [33] and the Scale for the Assessment of Negative Symptoms [34], respectively. Global scores on each domain (i.e., hallucinations, delusions, bizarre behavior, formal thought disorder, affective flattening, alogia, avolition, anhedonia) were used.

Clinical notes recorded in a standardized reporting system were reviewed to determine whether participants had been admitted to a hospital immediately prior to the onset of treatment.

### Quantitative data analysis

#### Preliminary analyses

Descriptive statistics for each variable were computed. Variables were transformed using logarithmic or square root transformations when skewness was present. Bonferonni-corrected one-way ANOVAs were conducted to determine if responses differed with respect to language of completion (i.e., English vs. French measures); and if responses on the youth resilience measure differed from responses on the adult resilience measure.

Multicollinearity among predictor variables was assessed through an examination of the correlations between variables and VIF statistics [35, 36].

#### Univariate analyses

Bonferonni-corrected univariate analyses were conducted to determine if predictors (the negative impact of FEP, positive reframing, spiritual coping, perceived social support, resilience, and recovery) and covariates (age, gender, time since diagnosis, symptoms of psychopathology and being hospitalized for FEP) were associated with PTG Inventory scores using Pearson’s and Spearman’s correlations.

To determine if the negative experience of psychosis had a curvilinear relationship with PTG, we tested if the quadratic term of the Subjective Experiences of Psychosis Scale scores predicted PTG Inventory scores. This entailed squaring the Subjective Experiences of Psychosis Scale scores; entering both the original and squared scores into two separate blocks in a hierarchical regression analysis; and determining if the change between blocks was significant.

#### Multivariate analysis

A hierarchical linear regression was conducted to determine if the main predictors and covariates predicted PTG. As per our protocol [14], all *main predictors* were evaluated in this model. Due to power considerations, only the *covariates* that were significantly related to PTG in the univariate analyses were included in the model. Significant covariates were entered into the first block, followed by the main predictor variables in a second block.

### Qualitative data analysis

Qualitative data were analyzed using thematic analysis [37]. Each transcript was checked for accuracy by two researchers independently, and conflicts were resolved through consensus. Transcripts were read several times to develop an overall understanding of the data; and subjected to open, line-by-line coding. Codes were reviewed and grouped into focused codes, categories and themes, which were checked against the original transcripts by the first author. In addition, the study team regularly discussed the codes, categories and themes to enhance the depth and rigour of the findings. An inductive approach favoring semantic level coding (i.e., reflecting participants’ descriptions of experiences) was used early on in the analysis. However, the analysis was also informed by theoretical models of positive change [38–40] and latent constructs (i.e., reflecting the underlying meaning and structure of themes). The final thematic map was developed by connecting themes, memos and notes with the larger narratives derived from participants’ interviews. Analytical memos and reflexive notes kept throughout the process also informed the analysis.

### Mixed methods data analysis

Once the qualitative and quantitative data were analyzed, convergence and divergence between findings was described using a weaving method [41] in the discussion section [42].

## Results

### Participants

One hundred forty-seven service users were approached according to the eligibility criteria defined above, 36 declined, and 111 participated. Data from 16 participants were omitted (i.e., they did not return questionnaires, did not properly complete questionnaires, or completed pilot versions of the questionnaires) and one participant withdrew consent from the study, yielding a final sample of 94. 14 service users, who were identified by their treatment team as having experienced positive change following FEP, were approached for participation in the *qualitative* component. Thirteen completed interviews, one declined, and one participant withdrew consent from the study, yielding a final sample size of 12. Participants who were interviewed also completed the questionnaires. Demographic and clinical characteristics of participants are shown in Table 1.

**Table 1:** Baseline demographic and clinical characteristics of study sample

Variable	Participants who were interviewed (*n* = 12)	All participants who took part in the study (*n* = 94)
	*f*(%)/*M(SD)*	*f*(%)/*M(SD)*
Age	24.27 (2.76)	25.52 (5.11)
Sex (female)	5 (41.6%)	40 (43.5%)
Education (at least high school)	11 (91.7%)	64 (76.2%)
Relationship status (in a relationship)	3 (25%)	9 (10.1%)
Visible Minority (yes)	7 (58.3%)	37 (45.7)
Born outside Quebec (yes)	7 (58.3%)	31 (35.2%)
Socioeconomic Status (middle to upper class)	9 (75.0%)	26 (38.8%)
Income derived from paid employment (yes)	5 (41.7%)	19 (24.1%)
Living with friends or family (yes) vs independently	11 (91.6%)	81 (96.4%)
Diagnosis		
*Schizophrenia Spectrum*	6 (50.0%)	48 (64.9%)
*Affective*	6 (50.0%)	26 (35.1%)
Baseline substance abuse (yes)	4 (33.3%)	27 (34.6%)

### Quantitative results

Descriptive statistics of the study variables are shown in Table 2. PTG Inventory scores did not differ between participants who completed the adult version of the resilience measure and those who completed the youth version. Responses on English and French questionnaires were not different and were not correlated with scores on the PTG Inventory.

**Table 2:** Descriptive statistics for predictor variables and their correlations with total Posttraumatic Growth Inventory Scores

Items	M/SD; n/***%***	Min – Max Possible Score	Correlation with Total Posttraumatic Growth
**Original Predictors**			
Negative impact of psychosis	41.42 (30.18)	0–116	.03
Spiritual coping	2.25 (2.04)	0–6	.52**
Positive reframing	3.24 (1.80)	0–6	.51**
Perceived Social support	5.45 (1.32)	1–7	.45**
Resilience	19.38 (3.62)	0–24	.42**
Recovery	164.98 (23.60)	1–205	.51**
**Additional Covariates**			
Age	25.35 (4.89)	–	0.13
Gender (female)	38 (41.3%)	–	0.10
Time since diagnosis (months)	20.1 (13.59)	6–75	−.03
Hallucinations	.45 (1.09)	0–5	−.08
Delusions	.57 (.90)	0–5	−.10
Bizarre behavior	.57 (.10)	0–5	.04
Positive formal thought disorder	.18 (.49)	0–5	−.02
Affective flattening	1.07 (1.22)	0–5	−.10
Alogia	.65 (.92)	0–5	.17
Avolition – apathy	1.86 (1.39)	0–5	.002
Anhedonia - asociality	1.67 (1.36)	0–5	−.09
Hospitalization for psychosis (yes)	55 (58.5%)	–	.23*

*Note.* * = *P* < .05; ** = *P* < .001

Multicollinearity among the predictor variables was not detected through correlational analyses (Table 3) or tolerance statistics (Range = .59–.83).

**Table 3:** Correlations among main independent variables

	1	2	3	4	5	6	7
**1.** Hospitalization	–	−.37**	−.01	−.16	.13	.06	.15
**2.** Negative impact of FEP	−.37**	–	.03	.07	−.14	−.08	−.14
**3.** Positive Reframing	−.01	.03	–	.32**	.35**	.37**	.27**
**4.** Spiritual Coping	−.16	.07	.32**	–	.22*	.23*	.20
**5.** Social Support	.13	−.14	.35**	.22*	–	.47**	.52**
**6.** Recovery	.06	−.08	.37**	.23*	.47**	–	.51**
**7.** Resilience	.15	−.14	.27**	.20	.52**	.51**	–

*Note.* * = *P* < .05; ** = *P* < .001

#### Univariate analyses of predictors

Spiritual coping, positive reframing, social support, resilience, and recovery were positively correlated with PTG Inventory scores (Table 2). Neither the linear nor the quadratic functions of the negative impact of FEP were significantly related to PTG Inventory scores.

#### Univariate analyses of covariates

Of the covariates, only being hospitalized upon entry into treatment was positively correlated with PTG Inventory scores (Table 2).

#### Multivariate analysis

The regression model contained hospitalization for FEP in one block and the six hypothesized predictors in a second block (the negative impact of FEP, resilience, perceived social support, spiritual coping, positive reframing, and recovery). The original, linear negative impact of FEP variable was used.

The first block containing hospitalization for FEP (β = .25, *P* = .021) was significant *F* (1,83) = 5.56, *P* = .02 and associated with a small proportion of variance in PTG Inventory total scores (R^2^adj = .05). Adding the main predictors in the second block was associated with a significant change in variance; R^2^change = .45, *P* < .001. This final model was significant *F* (7,83) = 11.83, *P* < .001 and explained 47% of variance in PTG Inventory total scores (R^2^adj = .47). Spiritual coping (β = .29, *P* = .001), positive reframing (β = .23, *P* = .01), recovery (β = .26, *P* = .009), and being hospitalized for FEP (β = .29, *P* = .001) were significant variables in this model (Table 4).

**Table 4:** Results of hierarchical linear regression examining predictors of positive change

Variable	β		SE β		Standard β	*P*	− 95%CI	+ 95%CI
**Block 1**								
Hospitalization for FEP	13.52		5.73		.25	.021	2.12	24.93
*R*^*2*^*change =* .06								
**Block 2**								
Hospitalized for FEP	16.15		4.73		.29	.001	6.73	25.57
Negative impact of FEP	.14		.08		.15	.08	−.02	.29
Resilience	.74		.72		.10	.30	−.69	2.17
Social support	1.88		1.99		.09	.35	−2.09	5.86
Spiritual coping	3.78		1.11		.29	.001	1.56	6.00
Positive reframing	3.46		1.31		.23	.01	.854	6.06
Recovery	.29		.11		.26	.009	.07	.50
*R*^*2*^*change =* .45								

### Qualitative results

**Fig. 1:**
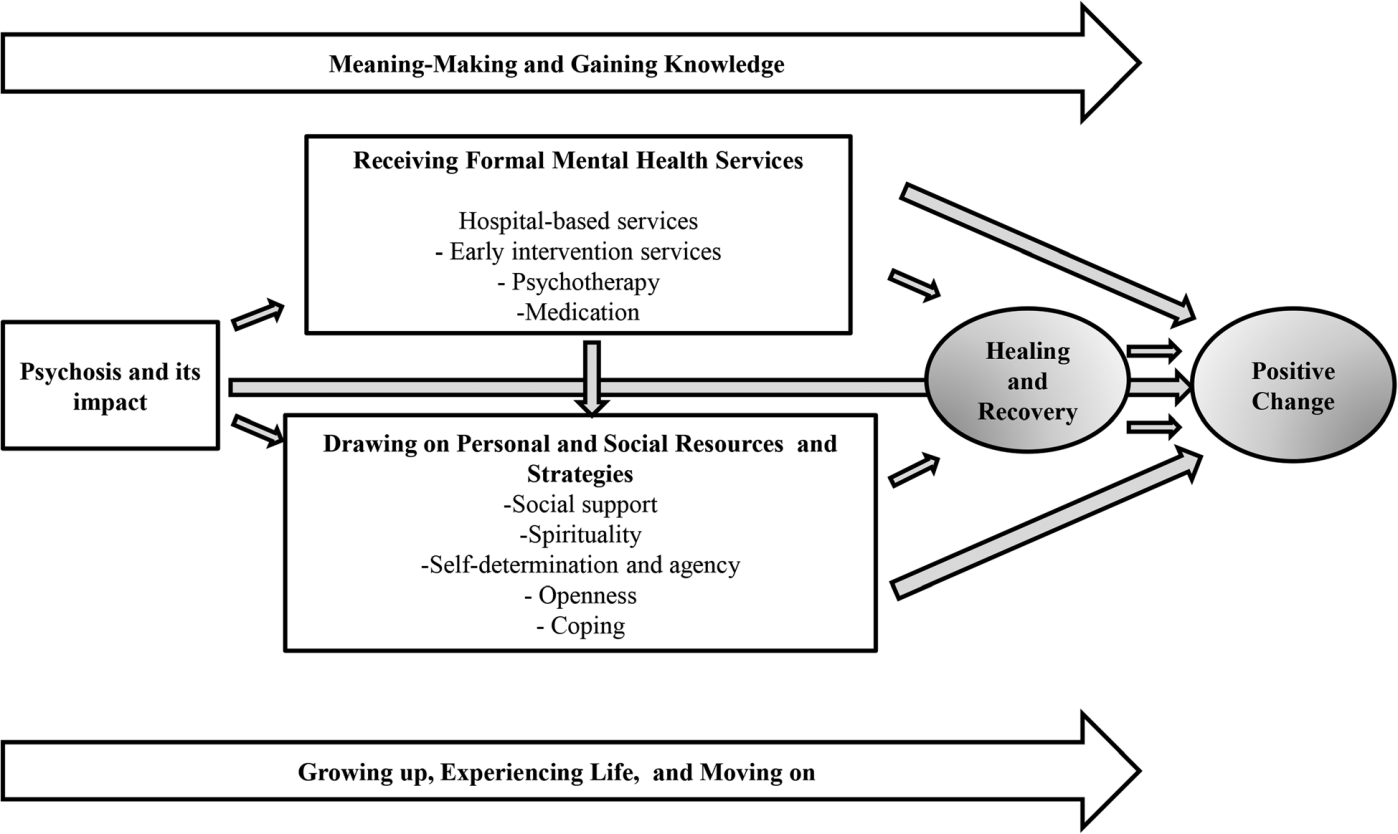
Qualitative findings depicting the perceived facilitators of positive change

Participants described a process involving various elements culminating in positive change. However, this process was not always linear. Participants perceived that the onset of psychosis was an important catalyst for positive change. Receiving mental health services, and drawing on personal and social resources and strategies to deal with their psychosis, were variously described as directly facilitating positive change or as facilitating recovery, which in turn facilitated positive change. Throughout this process, participants attempted to make sense of their experiences and sought information that they could use to strengthen the positive changes they experienced. Finally, participants described how growing up, maturing and life experiences unrelated to the FEP also led to positive change (Fig. 1).

#### Theme: psychosis and its impact

The first episode of psychosis was perceived as powerful enough to alter nearly every aspect of participants’ lives. Participants described impacts of FEP which made them re-evaluate their lives and chart new directions, including becoming disorganized, overwhelmed, and confused; having one’s “assumptive world” challenged and constantly asking oneself “why me?”; losing sleep, feeling depressed, hopeless, broken down, terrified or traumatized. For instance, when asked about what brought upon the changes they experienced following their mental health challenge, one participant noted:

Respondent: I think it’s the experiences that come with psychosis, the situation I was in because of the psychosis led to all these changes, you know? The uh situations it had caused (Mixed African and White European origins; schizophrenia-spectrum diagnosis, unemployed).

The fear of experiencing a second psychotic episode was also seen as an impetus to make important life changes, as illustrated by one participant who was reflecting on past habits:

Respondent: Yeah. I’m scared that I’m just going to get – like I’m just going to take a lot of coke, and I’m just going to be like – there’s going to be voices (White European origins, affective psychosis diagnosis, employed).

#### Theme: receiving mental health services and treatments

Following the onset of psychosis, participants received mental health services which were described as key facilitators of positive change. Importantly, many participants felt that being hospitalized was a key *turning point* during which they first realized that they needed to make changes in their lives. For instance, when asked about *how* psychosis led to experiencing change, one participant noted:

Respondent: Because the stress led me to be in the hospital, because I used to stress a lot. So that’s what happened - that’s how I got my meltdown and how I had a psychosis. So after I went home, back home from the hospital, I was still stressed out, I wasn’t myself completely, but I realized that because I stress too much it could come back, so I needed to make some changes in my life, so I needed to let go of certain stuff, so I could feel better and I could be a better person (South Asian origins; affective psychosis diagnosis; employed).

The care participants received at the early intervention service was also seen as facilitating positive change. Important elements of such care included having caring, flexible case managers who treated participants like “normal” people and believed in their potential. Aspects of treatment that were aligned with therapeutic alliance, such as emotional support, were emphasized as particularly important. Likewise, the willingness and ability of staff to listen to participants was highlighted. Instrumental aspects of care that were perceived as facilitating positive change included help with finding housing, going back to school or work, achieving broader life goals, and meeting new people through organized group activities. This also included addressing the broader economic and social conditions affecting their mental health. For instance, when asked about the role staff of the early intervention service played, one participant responded:

Respondent: They listen well and they’re right there, when I was having a really rough time, my psychiatrist saw me twice a week, when I was super suicidal, and they were thinking of putting me in the hospital, maybe, but then they trusted my family to be able to watch me and to be able to make sure that I didn’t like hurt myself, and just being able to recognize and acknowledge the situation that I’m in, in the context that I’m in, they were really flexible (Mixed African and White European origins; schizophrenia-spectrum diagnosis; unemployed).

An additional important aspect of care that was perceived to facilitate positive change was psychotherapy, which helped participants cope with their problems (e.g., by seeing negative experiences in a positive light) and better handle their emotions, as illustrated by the following quote:

Interviewer: So you think CBT [cognitive behavioural therapy] really helped?

Respondent: It really helps. It really helps me think, see a solution for things. And for a problem, and let’s say a problem happens ... like now, I don’t think oh, I don’t have anything ... there’s nothing I can do. Yes, there is something I can do. Right now I’m thinking yes, there is something that I can do to improve my situation and to not be stuck to the feeling of being stuck, unless it’s lasting (Chinese origins; schizophrenia-spectrum diagnosis; unemployed).

While many participants struggled with the side effects of medication, they also described how medication fostered improved mental health as well as positive change.

Interviewer: So what do you feel has like helped you to get to where you are now?

Respondent: My medication that they threw me on. Yeah because if I didn’t take the medication I’d probably still be the same person I was (White European origins; affective psychosis diagnosis; employed).

In contrast, another participant felt that taking medication was only useful in laying the foundation for her psychological stability; but that taking too much medication may have prevented her from experiencing positive change.

Respondent: I took medication twice in the hospital, I’m not going to take anymore, because…I just feel like I have to face – and that if I just take medication I deny the deeper meaning of what’s going on and what’s happening and why.

Interviewer: And the deeper meaning was bringing you into your authentic self and then challenging how you were before?

Respondent: Exactly. Challenging how I was before, exactly (White European origins; affective psychosis diagnosis; unemployed).

#### Theme: Drawing on personal and social resources and strategies

Participants drew on several resources and strategies to facilitate positive change. The most important such resource was social support offered by their treatment team, family, friends, peers with lived experience of psychosis, and romantic partners. For instance, when asked about the support received by one participant’s family, they replied:

Respondent: Well, they’ve been giving me support financially. They’ve been supporting me emotionally, emotionally, like if I need help with anything, if I’m down they are always there. Even when I was in [city name] like they were very worried. You know, wondering like what’s going on. Um, at one point my mother even came down to [city name] to see what was going on with me, you know? So all these things made me feel like my family really love me, you know? So it makes me feel like living each day, not only for myself but for them as well. (African origins, affective psychosis diagnosis; unemployed]

Participants’ loved ones facilitated positive change by “being there”; treating participants like “normal” rather than sick people; engaging with participants in everyday activities; listening to participants and providing opportunities to express their feelings; and providing instrumental support (e.g., financial support):

Respondent: They listened to me, they encouraged me to just - they always told me that it’s a temporary thing - I’m going to get through it - it’s not permanent. They would take me out just to like - they were really patient with me and they will really encourage me to do better and not to feel like I’m stuck in this, because I felt stuck (South Asian origins; affective psychosis diagnosis; employed).

In addition, two participants described being supported by voices or visions of loved ones, such as deceased relatives.

Interviewer: Do you feel like you have people in your life that support you, through your experiences and when times are difficult?

Respondent: Being honest, not really. But it’s like they support me ... I don’t know. Not in real life because we don’t talk with each other, but I feel like they support me in the other world (African origins; schizophrenia-spectrum diagnosis; unemployed).

Spirituality and religion were seen as important resources that facilitated positive change. Illustratively, one participant felt that she had become kinder to others following FEP:

Respondent: I do believe in God - I believe there’s a bigger - a higher spirit or a - I don’t know how to call it - just God…And I just feel like to be kind in life, doing good for others, doing good for yourself, is a good way to live life. (South Asian origins; affective psychosis diagnosis; employed).

Others described how engaging in spiritual or religious practices (e.g., meditation), attending religious institutions (e.g., church), or feeling loved by God facilitated recovery and, by extension, positive changes, as illustrated by the following quote:

Interviewer: Has the church and that kind of spiritual stuff, does that also help you?

Respondent: Yes, a lot.

Interviewer: In what way?

Respondent: To be calm and every day to… do what I have to do, but to leave some stuff to God, that I cannot control everything. (Latin American origins; affective psychosis diagnosis; employed)

Some described how their positive changes were facilitated by ongoing spiritual experiences. For instance, some described having new knowledge revealed to them by the voice of God. Perceptions that ongoing spiritual experiences facilitated positive change depended on participants’ belief in the veracity of such experiences. For instance, when reflecting upon the new spiritual insights he had gained through his mental health challenge, one participant noted:

Respondent: I put all my entourage in what I read and it makes so much sense. So sometimes, sometimes it even tells me like what’s going to happen tomorrow, and it really happens. So that’s how it changed really – my perspective about life is that the word of God is so important in life (African origins; schizophrenia spectrum diagnosis; unemployed).

Participants also relied on their own agency and self-determination to facilitate positive change. For some, this involved taking risks needed to improve their lives. Others described the importance of having the strength and willpower needed to make positive changes; believing in themselves and being honest with their feelings; and making conscious decisions to change for the better. Some also described how being authentic to their values and passions helped them live more “congruent” lives. Illustratively, when reflecting on what factors may have led to the changes they experienced, one participant noted:

Respondent: I know that it [psychosis] makes me a lot more aware, it makes me a lot more stronger. I feel like I’m stronger now just because. I had to fight to stay alive. Right? I had to fight my depression in order to not commit suicide. Which I’ve attempted. I had to convince myself that I don’t deserve to inflict pain on myself. I have to really, really love what I’m doing with every second of my life because if I don’t, then I’m wasting my time (White European origins; schizophrenia-spectrum diagnosis; unemployed).

In addition, some participants described how being open to change and willing to learn from challenges led to self-improvement, as reflected by the following quote:

Interviewer: Okay. So back to the original question. What do you feel has influenced – you said psychosis was the ultimate catalyst, but you were about to say something about what else had influenced all these changes?

Respondent: I’ve always had that desire to improve myself and I’ll always been very open to criticism, that uh positive criticism, you know, anything that could help me be a better person, I’ll always take it (Mixed African and White European origins; schizophrenia-spectrum diagnosis; unemployed).

Participants also used coping strategies to deal with FEP and its aftermath, which in turn facilitated positive change. Such strategies included finding ways to disengage from stressful situations; relinquishing behaviors or activities that were seen as harmful after the onset of psychosis; or learning to “get a grip” on “life’s curveballs”.

Interviewer: What role do you feel that stopping drugs played in getting to where you are now?

Respondent: A big role because if I didn’t stop drugs I would probably still be on the path I was on. Yeah (White European origins; affective psychosis diagnosis; employed).

#### Theme: Healing and recovery

Drawing on formal mental health services as well as personal and social resources and strategies helped participants heal and recover from the FEP. For some, “clinical” recovery (and not the psychosis) was seen as the *main* facilitator of positive change. When asking for clarification on what the main driver of changes they experienced could be, one participant adamantly clarified that:

Respondent: All of my positive changes came from not those things, but getting out of those things. That is something I will absolutely make clear is that none of the positive effects that I have in my life right now came from these awful, crappy, terrible diseases (White European origins, schizophrenia-spectrum diagnosis; unemployed).

Participants felt that they were at varying points in their recovery. Some felt like they were still healing while others described feeling fully recovered. Some felt their recovery was synonymous with positive change and a new way of being. One participant reflected that recovery, as defined as a return to normality, was not possible:

Interviewer: Where do you feel you are in the recovery process...what [does] that even mean to you?

Respondent: I don’t know what it means anymore, because there’s no ... like someone said like, there’s a new normal, kind of, like there’s no going back to normal cause like there’s no way back. It’s just moving forward and finding the new way that I am and accepting the new way that I am…. Yeah, I’m not really going to recover, and I don’t really want to go back to where I was before, at all, cause like it didn’t work out very well for me, where I was headed (Mixed African and White European origins; schizophrenia-spectrum diagnosis; unemployed).

#### Theme: Making meaning and gaining knowledge

Participants described how they engaged in a meaning-making process that facilitated positive change. This process began when participants first became unwell and assigned causal attributions to their mental health problems, which included seeing psychosis as a positive, useful, therapeutic or spiritual experience; as well as viewing psychosis as a medical problem explainable by science. Some also described how psychosis provided meaningful information that contributed to self-improvement. For instance, when reflecting upon reasons why they no longer wished to take antipsychotic medication, one participant said:

Respondent: It’s [psychosis] information. I feel like I’m in a stage where I’m able to receive information about what’s really going on with me and then like now I’m out of that state, right, so now I have to take that information that I was receiving and try to apply it to reality (White European origins; affective psychosis diagnosis; unemployed).

In addition, participants appraised and reflected upon aspects about themselves and their lives in the aftermath of psychosis. A key facet of this process involved experiencing important realizations about the various aspects that participants felt needed changing. Such realizations were described as fundamental to many of the positive changes that participants experienced. Illustratively, when one participant asked about what contributed to their improved emotional well-being following FEP, they said:

Respondent: I let go of that because I realized I can’t control what they’re doing, what, or what they do, or what mistakes they make because I can’t fix it for them. The only thing I can fix is myself. So I let go of that, so. That in a way I’m better because I don’t stress about those things anymore (South Asian origins; affective psychosis diagnosis; employed).

A third meaning-making process involved consciously searching for ways that positive change could follow FEP or other life experiences (i.e., positive reframing, which for some was seen as facilitated through psychotherapy), as illustrated by the following quote:

Respondent: Like, I used to see, you know, okay, this was bad, but how can I find the good in it and. .. But it’s like now that I look back, it’s [psychosis] just all inherently positive, because it gives me so much each time (White European origins; affective psychosis diagnosis; unemployed).

In addition, some drew on cultural frameworks describing growth following adversity which helped participants understand the nature of psychosis, why it happened, and how they changed following their psychosis. Such frameworks included life mottos; religious or spiritual norms; descriptions of spiritual emergencies; and the lives of pop culture figures, as illustrated by the following example:

Interviewer: Yeah, just tell me as much as you can about that [How they felt stronger following the FEP]. Like how were you before the psychosis?

Respondent: Well, it’s kind of like the quote that says, like when you fall, you fall, but when you rise, you become stronger, you know? So you kind of like – it’s kind of like my motto to my life (White European origins; affective psychosis; employed).

Several participants searched for information about and gained new knowledge related to the domains in which they experienced positive change. For instance, one participant who gained spiritual knowledge following FEP engaged in extensive reading of religious texts to further develop this knowledge. Another participant who felt that his psychosis had made him a better activist spent time reading and learning about social justice to further solidify his role as an activist:

Interviewer: Aside from the psychosis itself, do you feel like there are other things that might have played into your being a better activist?

Respondent: I’m in a lot of spoken word scenes, and I must spend hours and hours every couple days just…reading and going through, different activist’s art and understanding it. I’m not saying that psychosis is the only reason for me to be an activist, I just feel like without it I wouldn’t have the experiences that I do (White European origins; schizophrenia-spectrum diagnosis; unemployed)

#### Theme: Growing up, experiencing life, and moving on

Some participants described how additional, important life events unrelated to psychosis facilitated positive change. Such experiences included being far from home and needing to survive; having a loving romantic partner who motivated change; and making and learning from mistakes and life choices. For example, when asked about the changes experienced following FEP, one participant reflected:

Respondent: And so I like I kind of had this thing with my parents, this opposition I don’t how we call it, but it’s like, there’s a moment where you need more space as a child because you grow up and the parents like have difficulty giving that space (Arab origins; schizophrenia-spectrum diagnosis; unemployed).

In addition to life experiences, many described how their positive changes arose from growing up. Some—especially those whose mental health problems emerged at a young age— described how their positive changes were facilitated by completing a life phase or attaining a milestone; by becoming more mature; or through a desire to become more mature. These participants had difficulty unraveling the extent to which their positive changes were due to FEP versus growing up.

Interviewer: Okay. Has psychosis changed your perspective on life?

Respondent: Yes, I think so. Yes.

Interviewer: In what way?

Respondent: Well, it’s hard to say if it was psychosis or just life experiences growing up (Mixed African and White European origins; schizophrenia-spectrum diagnosis; unemployed).

## Discussion

This study investigated predictors and perceived facilitators of PTG and positive change following FEP using a mixed methods convergent design. The quantitative and qualitative findings both diverged and converged in several ways.

### Convergence between quantitative results and quantitative findings

#### Spirituality/religion

Both sets of findings reveal how positive change was facilitated by spiritual coping, which is consistent with other studies on PTG [43]. This finding highlights the potential role that spirituality and religion play in buffering people from stress [44], and supports theoretical claims that spirituality or religion “can provide a unifying philosophy of life” [45] (p. 738) that can help persons grow from adversity [39]. Other studies have shown that “positive” spiritual or religious coping (e.g., seeking spiritual support) is a stronger predictor of positive change than “negative” spiritual or religious coping (e.g., experiencing religious struggles) [46]. Spiritual coping often fell under such a “positive” definition in the qualitative findings.

#### Positive reframing

Positive reframing was an important facilitator of positive change in both sets of findings, which is consistent with our systematic review’s earlier finding [4], and with studies of PTG following other adversities [47]. Our findings support claims that positive reframing is an important mechanism through which persons ascribe a constructive meaning to adversity [48].

#### Recovery

Both quantitative and qualitative findings revealed that subjective recovery was a key facilitator of positive change. This finding is consistent with other studies showing a link between recovery and positive change [11, 49], and suggests that experiencing broader areas of subjectively defined improvement are needed to grow following FEP, or that recovery and positive change may be similar processes. Relatedly, some participants described how they viewed their “psychotic” experiences as strengths that facilitated positive change. This finding is consistent with studies showing how voices can be useful or serve a purpose [50].

#### Hospitalization

Being hospitalized for FEP facilitated positive change in both sets of findings, which is consistent with one study describing how some participants experienced positive change through seeking help, and eventually being hospitalized for FEP [32]. This finding may reflect the distressing or traumatic nature of being hospitalized for FEP for the first time, especially if such hospitalizations were involuntary [51]. To our knowledge, this finding has not been observed following other adversities. Our qualitative findings also revealed that being hospitalized was both distressing and an important turning point in participants’ lives, which is consistent with studies in the context of other adversities [7].

### Divergence between quantitative results and qualitative findings

#### The negative impact of FEP

The negative impact of FEP did not predict PTG in the quantitative results. Yet, the onset of psychosis and the negative and difficult events it was associated with were seen as important in the qualitative findings. This divergence may reflect the measure we used to assess the negative impact of psychosis [22]. Many studies reporting a relationship between the impact of an adversity and PTG employ measures of the emotional intensity of an adversity [7], which was not the primary focus of the measure we employed.

#### Resilience and social support

In the quantitative component, resilience and social support did not predict PTG in the multivariate analyses. In the qualitative component, participants spoke of how drawing on personal and social resources (which fall under the concept of resilience) facilitated positive change. Further, social support was seen as a key social resource which facilitated positive change.

This divergence may stem from how resilience and social support were measured in the quantitative component. The resilience scale measures facets that may not contribute to PTG. Similarly, our measure of social support [24, 52] only captured perceptions of *who* offered support, and not the *ways* that social support could be offered, which was described in the qualitative findings and may have been a stronger predictor of positive change. This divergence may also reflect the purposive sampling of participants for the qualitative component.

#### Mental health services and treatments

Participants described how receiving mental health services and treatments facilitated positive change, a process which was not measured in the quantitative component. This finding echoes results of other studies which showed that humane and normalizing mental health care was an important facilitator of positive change [4]. This finding suggests that approaches to care that are consistent with established guidelines for recovery-oriented care for mental illnesses [53], and with the foundational philosophy of early intervention services (i.e., that they are based on fostering hope and optimism) may be key in shaping positive change. The finding that psychotherapy facilitated positive change is also consistent with previous work [54], and points to the capacity of psychotherapists to foster positive change among service users [55]. Medication was seen as facilitating healing, recovery and positive change in the qualitative findings, which is consistent with our systematic review [4]. This has also been reported in a few studies of other illnesses where receiving treatment contributed to positive change [56].

#### Meaning-making and gaining knowledge

The qualitative findings diverged from the quantitative results in revealing how participants engaged in meaning-making processes that facilitated positive change. This finding is consistent with other studies showing that meaning making predicts PTG in non-psychosis samples [57, 58], and support models depicting the search for meaning as a key facilitator of positive change [39, 48]. This finding is consistent with our systematic review [4]. Similarly, the qualitative findings revealed that some positive changes were facilitated through gaining knowledge about the domains within which positive change was experienced. Prior work has also demonstrated the value of learning and receiving educational instruction in contributing to transformational experiences [59].

#### Normative developmental processes

Normative developmental processes were seen as facilitating positive change, a nuance which was not captured in the quantitative results. This finding is seldom described in other studies [5]. This finding supports claims that developmental and adversity-related trajectories can share similar outcomes, and that multiple and potentially interacting paths to positive change exist [60]. Our finding may also be attributable in part to the young age of our sample.

#### Implications

This is the first empirical investigation of predictors or perceived facilitators of PTG and positive change following FEP to employ mixed methods. The findings suggest that individual (e.g., meaning making) and contextual factors (e.g., mental health services) may play a role in fostering growth following psychosis. Positive change should therefore not be viewed as a person’s sole responsibility; instead, multiple stakeholders aiming to foster growth have a role to play in facilitating positive change. For instance, family members should provide emotional and instrumental support; mental health services should be recovery-oriented, optimistic and normalizing; and policy makers should develop strategies to ensure important services that may facilitate positive change are properly funded [61].

Our findings should not be construed as romanticizing FEP or the hospitalization experience. Although it can be a significant turning point, both are often a traumatic experience [62], and efforts must always be taken to prevent FEP and reduce the need for hospitalization.

### Strengths and limitations

We conducted this study with a high level of methodological rigour while giving equal weight to the philosophical paradigms guiding the study. Our findings were able to capitalize on the strengths of both methods used, yielding a holistic understanding of the topic. Participants were a well-characterized sample of service users with FEP recruited from a single catchment area.

Due to the study design, it was impossible to establish if the independent variables had a causal relationship to positive change. Another limitation was that there was conceptual overlap, and some degree of measurement overlap between the independent variables used in this study. We relied on the treatment team to identify service users who had experienced positive change and could be approached for qualitative interviews. This may have resulted in oversampling service users with whom clinicians had a strong therapeutic alliance. Finally, although we followed established recommendations [15] and purposefully recruited participants until no new information was obtained at by the 12th interview, perhaps additional information would have been obtained had we recruited additional participants.

### Future directions

Future studies should explore positive change following psychosis in different contexts (e.g., hearing voices groups, peer support groups, etc.) to elucidate the importance of non-medical supports. Longitudinal studies of predictors of positive change, as well as studies using other qualitative methods (e.g., ethnography, etc.), are also needed.

## Conclusions

The quantitative results from this study revealed that being hospitalized for FEP, spiritual coping, positive reframing, and subjective recovery predicted PTG. The qualitative findings revealed that the experience of psychosis was perceived as directly motivating positive change, but also as leading participants to draw on formal mental health services and treatments and personal and social resources. Drawing on services and resources were perceived to facilitate positive change directly but were also perceived to lead to recovery and in turn positive change. Participants also described how they engaged in a meaning-making and knowledge-gaining process that facilitated positive change; and some reflected on how normative developmental processes may have contributed to positive change. The study results can help clinicians and policy makers identify ways to better support service users’ capacity to experience positive change in the aftermath of the onset of psychosis.

## Data Availability

The data cannot be shared or made openly available because we were not granted permission to do so by participants and the McGill University Ethics Board.

## Abbreviations

PTG: Posttraumatic growth
FEP: First episode psychosis
